# Supplementary material for: In vitro culture of freshly isolated Trypanosoma brucei brucei bloodstream forms results in gene copy-number changes
Source: PLoS Negl Trop Dis. 2021 Sep 13;15(9):e0009738. doi: 10.1371/journal.pntd.0009738 (PMC8459984; doi:10.1371/journal.pntd.0009738)
Supplement: S1 Text — (PDF) [file pntd.0009738.s001.pdf]

S1 text: Preliminary characterization

1. *T. brucei* isolates from cattle grown in mice

We inoculated and successfully grew 3 recently isolated *Trypanosoma brucei brucei* strains from cattle (Table 1) and made fresh stabilates.

Table 1

Isolate ID	Simplified name	Source Village/Parish	Source District	Date of Isolation
Tb065BAPC	MAK65	Bunya	Apac	01/02/2016
Tb236BAPC		Bunya	Apac	01/02/2016
Tb098AAPC	MAK98	Apuru	Apac	30/07/2016

For simplicity Tb065BAPC and Tb098AAPC are designated MAK65 and MAK98 in the paper, emphasizing their original characterization at Makerere University. References in this text are at the end of the text.

2. PCR characterization

Internal transcribed spacer (ITS)

We confirmed *Trypanozoon* status in all isolates by carrying out internal transcribed space (ITS) PCR, which yields a band size of approximately 480bp [1]. We confirmed that the parasites were *T. brucei brucei,* and therefore not infective for humans, by PCR for the *SRA* gene [2]. Human-infective *T. brucei rhodesiense* yield a product of 284bp. In both cases *T. b. rhodesiense* LW042 [3] was used as a positive control.

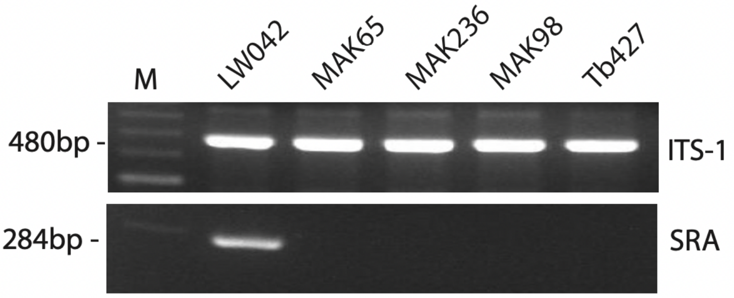

Fig 1: PCR confirmation that MAK65 and MAK98 are *T. brucei brucei*: positive PCR for ITS-1 and negative result for *SRA*.

Microsatellites

Genotyping of the *T. brucei* strains was carried out using microsatellites designated for *T. brucei* (Table 1) as described in [4].

The three recently isolated strains were again compared with *T. b. rhodesiense* human isolate LW042 (Fig 2). Tb236B and MAK65 were similar and could be distinguished from both MAK98 and LW042. This was probably because Tb236B and MAK65 were isolated from cattle in the same village/parish. Since we wanted to investigate different strains, Tb236B was not studied further.

Fig 2: Microsatellite analysis.

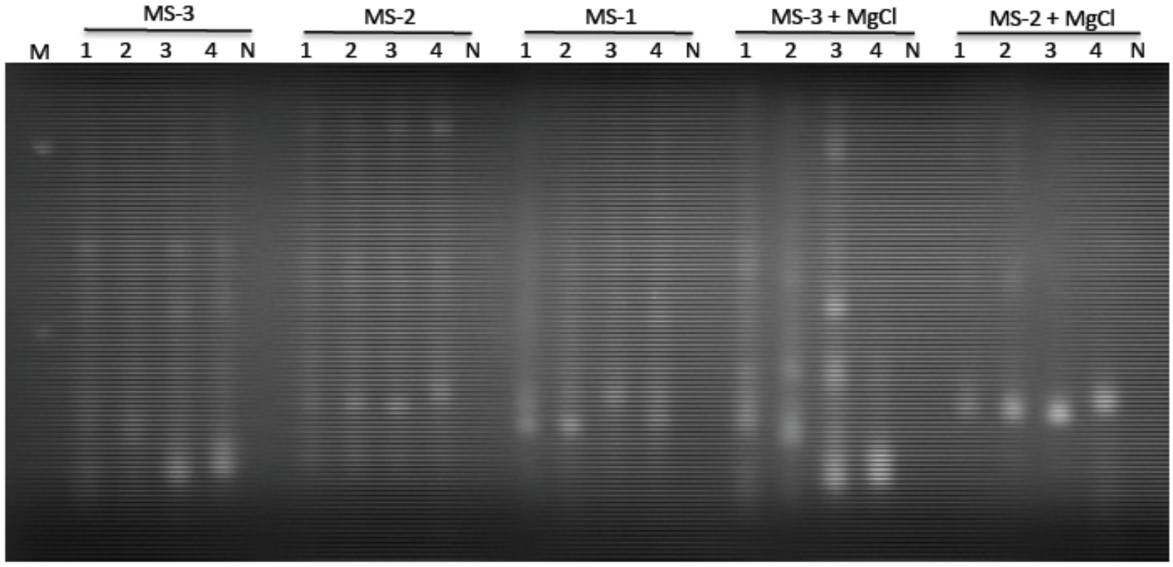

Microsatellite analysis of 1-Tb236B, 2-MAK65, 3-LW042, 4-MAK98 using the primers shown in Table 2.

Table 2

Locus (code)	Primer (outer)	Sequence (5'-3')	Primer (nested)	Sequence (5'-3')
Ch2/PLC (MS-1)	PLC-G2<br>PLC-H4	ttaagtggacgacgaaataacaaca<br>ttcaaacaccgtccccctcaataat	2/PLC-G<br>2/PLC-H3	caacgacgttggaagagtgtgaac<br>ccactgacctttcatttgatcgctttc
Ch4/M12C12 (MS-2)	M12C12-C<br>M12C12-B	aaacctcatccagtcgcactgg<br>taccctcatcaagtggtcg	M12C12-A<br>M12C12-D	tggacacacagaagcctaccg<br>agtgtggtggtgcgtgcaaacttgg
Ch5/JS2 (MS-3)	JS2-C<br>JS2-D	agtaatgggaatgagcgtcaccag<br>gatcttcgcttacacaagcggtac	JS2-AFAM<br>JS2-B	gattggcgcaacaactttcacatacg<br>ctttcttccttggccattgttttactat

References

1. Njiru Z, Constantine C, Guya S, Crowther J, Kiragu J, Thompson R, et al. The use of ITS1 rDNA PCR in detecting pathogenic African trypanosomes. Parasitol Res. 2005;95:186-92. doi: 10.1007/s00436-004-1267-5.
2. Radwanska M, Chamekh M, Vanhamme L, Claes F, Magez S, Magnus E, et al. The serum resistance-associated gene as a diagnostic tool for the detection of *Trypanosoma brucei rhodesiense*. Am J Trop Med Hyg. 2002;67:684-90.
3. Mulindwa J, Mercé C, Matovu E, Enyaru J, Clayton C. Transcriptomes of newly-isolated *Trypanosoma brucei rhodesiense* reveal hundreds of mRNAs that are co-regulated with stumpy-form markers. BMC genomics. 2015;16:1118. PubMed Central PMCID: PMC10.1186/s12864-015-2338-y, PMID: 26715446.
4. Kato CD, Alibu VP, Nanteza A, Mugasa CM, Matovu E. Population genetic structure and temporal stability among *Trypanosoma brucei rhodesiense* isolates in Uganda. Parasit Vectors. 2016;9:259. Epub 2016/05/05. doi: 10.1186/s13071-016-1542-1. PubMed PMID: 27142001; PubMed Central PMCID: PMCPMC4855840.
