# Supplementary material for: In vitro culture of freshly isolated Trypanosoma brucei brucei bloodstream forms results in gene copy-number changes
Source: PLoS Negl Trop Dis. 2021 Sep 13;15(9):e0009738. doi: 10.1371/journal.pntd.0009738 (PMC8459984; doi:10.1371/journal.pntd.0009738)

A. PAD1 staining of MAK65 from rat B

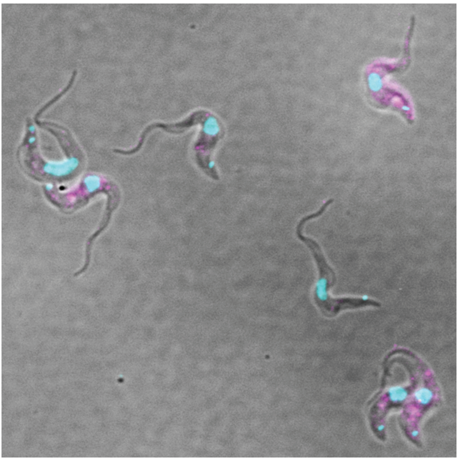

B. Stumpy-form differentiation *in vitro* vs *in vivo*

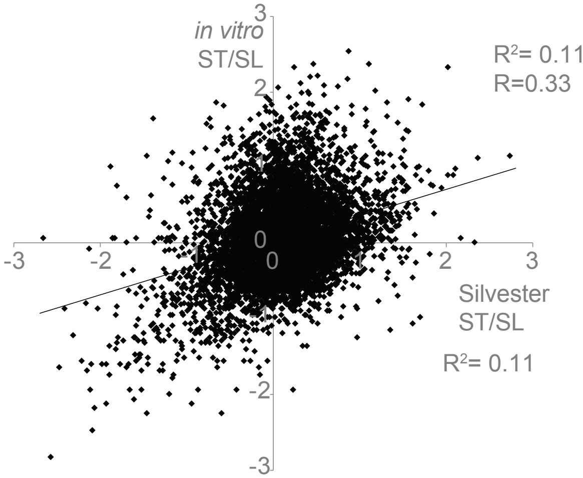

C. Poly(A) selection depletes long mRNAs

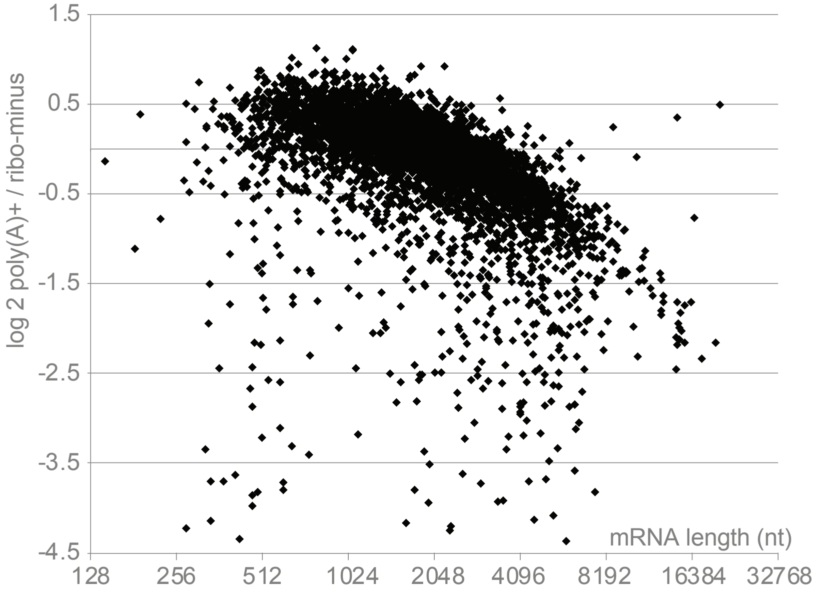

Supplement: S1 Fig — A. Example of PAD1 staining for sample 65A. PAD1 is in magenta and DNA is cyan. B. Comparison of published results for in vitro [23] and in vivo [22] stumpy differentiation. In each case the log2 ratio of stumpy-form to long-slender form EATRO1125 is shown. C. Total RNA from MAK98 trypanosomes (sample from rat A) was either selected on oligo d(T) to give poly(A)+ RNA, or treated with RNase H and oligonucleotides complementary to the rRNA in order to give ribo-minus RNA. The log2 ratio of poly(A)+ to ribo-minus was is on the y-axis and the annotated mRNA length (log scale) on the x-axis. Results are in S1 Table, sheets 2 and 5. (PDF) [file pntd.0009738.s002.pdf]
