# Supplementary figures and images for: In vitro culture of freshly isolated Trypanosoma brucei brucei bloodstream forms results in gene copy-number changes
Source: PLoS Negl Trop Dis. 2021 Sep 13;15(9):e0009738. doi: 10.1371/journal.pntd.0009738 (PMC8459984; doi:10.1371/journal.pntd.0009738)

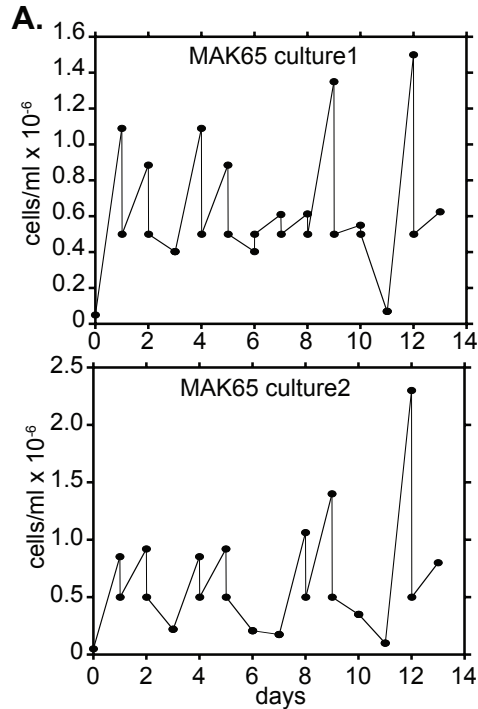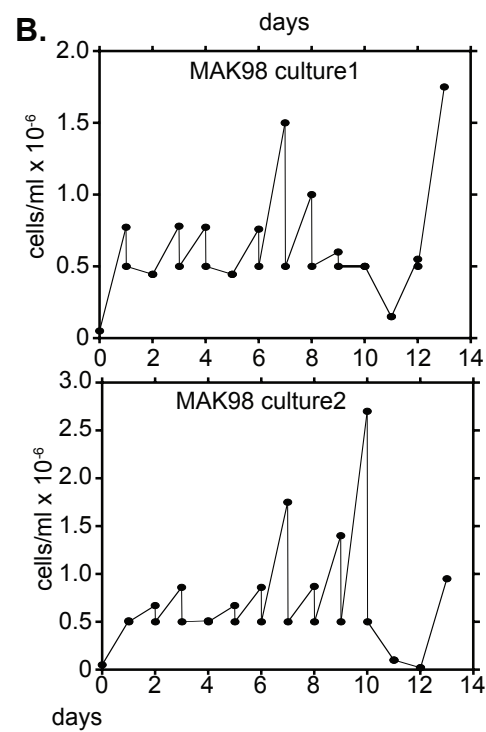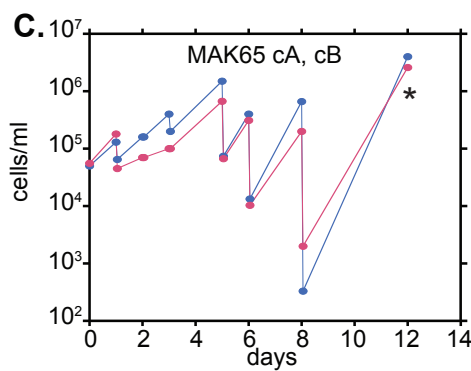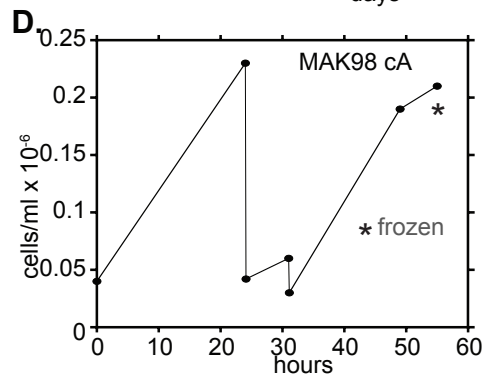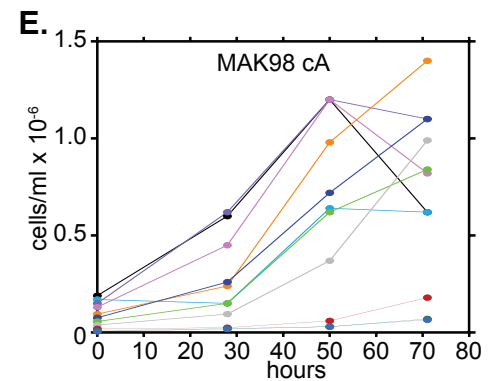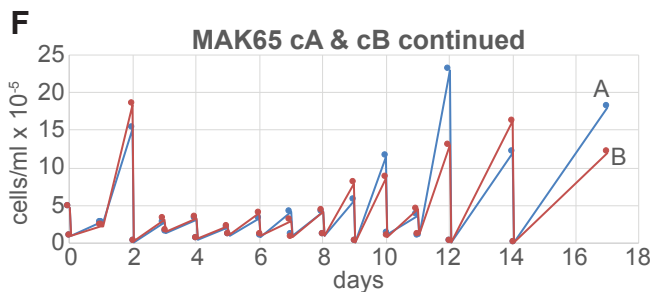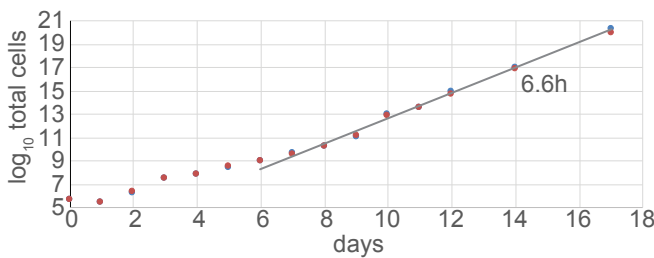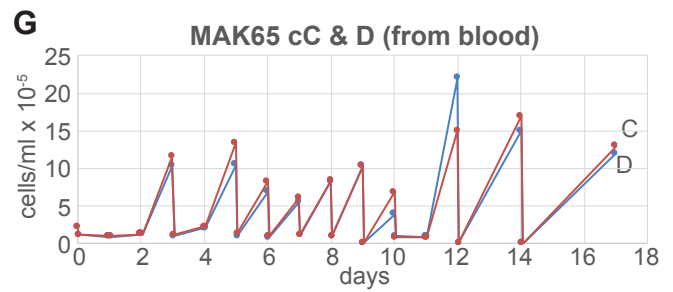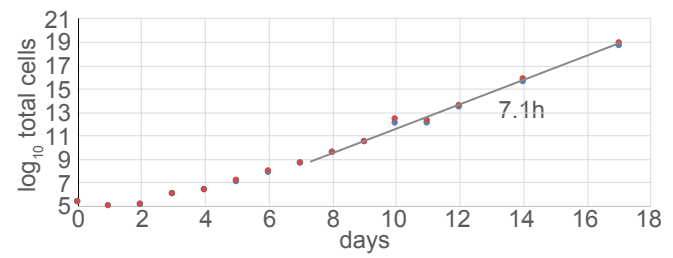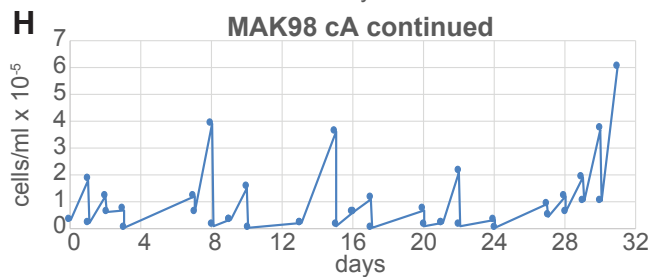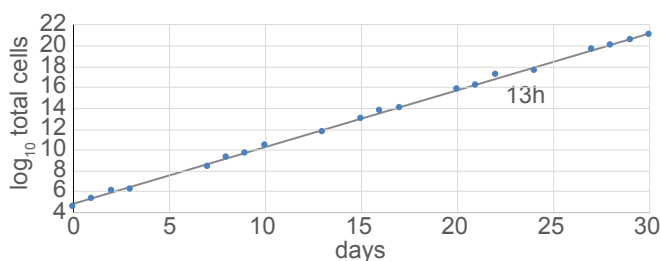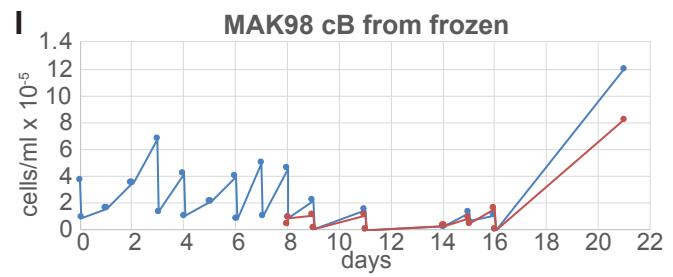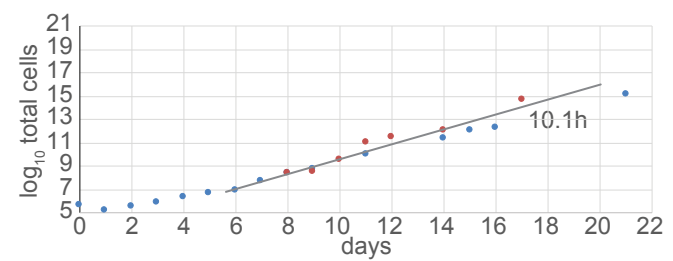

Supplement: S2 Fig — These data were used to make the cumulative curves in Fig 3; histories are in Fig 1. A. Initial cultures of MAK65, with parasite densities shown on a linear scale with dilutions. B. Initial cultures of MAK98, with parasite densities shown on a linear scale with dilutions. In each case the upper panel shows parasite densities on a linear scale and the lower panel shows cumulative parasite numbers on a log scale. The lines on the log scale graphs indicate the part used for division time calculations. In each case DNA was harvested at the end of the culture period, and stabilates were made. C. Cultures of MAK65 (cultures A and B) prior to creation of frozen stocks. D. Culture of MAK98 (A) prior to creation of frozen stocks. E. The MAK98 culture from (D) was subjected to serial dilutions in order to determine the maximum cell density. F. MAK65 cultures A and B (cA and cB) were continued from frozen stocks (panel C) to give a total culture time of 30 days. The upper panel is on a linear scale, and the lower panel is on a log scale showing the division time after 3 weeks of culture adaptation. G. MAK65 cultures C and D (cC and cD) were freshly initiated from blood stabilates. Details are as for (A) except that the culture time is shorter. H. Mak98 culture A (cA) was continued directly from panel D. I. Mak98 culture B (cB) was initiated from a frozen stabilate from Fig 2F. For genome analyses results from the two final cultures were >99% identical so the counts were pooled. (PDF) [file pntd.0009738.s003.pdf]
