# Supplementary material for: In vitro culture of freshly isolated Trypanosoma brucei brucei bloodstream forms results in gene copy-number changes
Source: PLoS Negl Trop Dis. 2021 Sep 13;15(9):e0009738. doi: 10.1371/journal.pntd.0009738 (PMC8459984; doi:10.1371/journal.pntd.0009738)
Supplement: S3 Fig — All reads for MAK65 starting population, and culture A, were allowed to align once to the TREU927 genome. The resulting mapped reads were visualized using the Integrated genome viewer (Broad Institute). The region surrounding the gene encoding the major cytosolic HSP70 is shown. The relative copy number for HSP70 can be seen by comparing its read density with that over the surrounding single-copy regions. A key is below the alignment and few differences between the genomes are highlighted; these show regions where there might have been selection for particular variants. Analysis of more independent cultures would be needed to identify the most significant selective events. (PDF) [file pntd.0009738.s004.pdf]

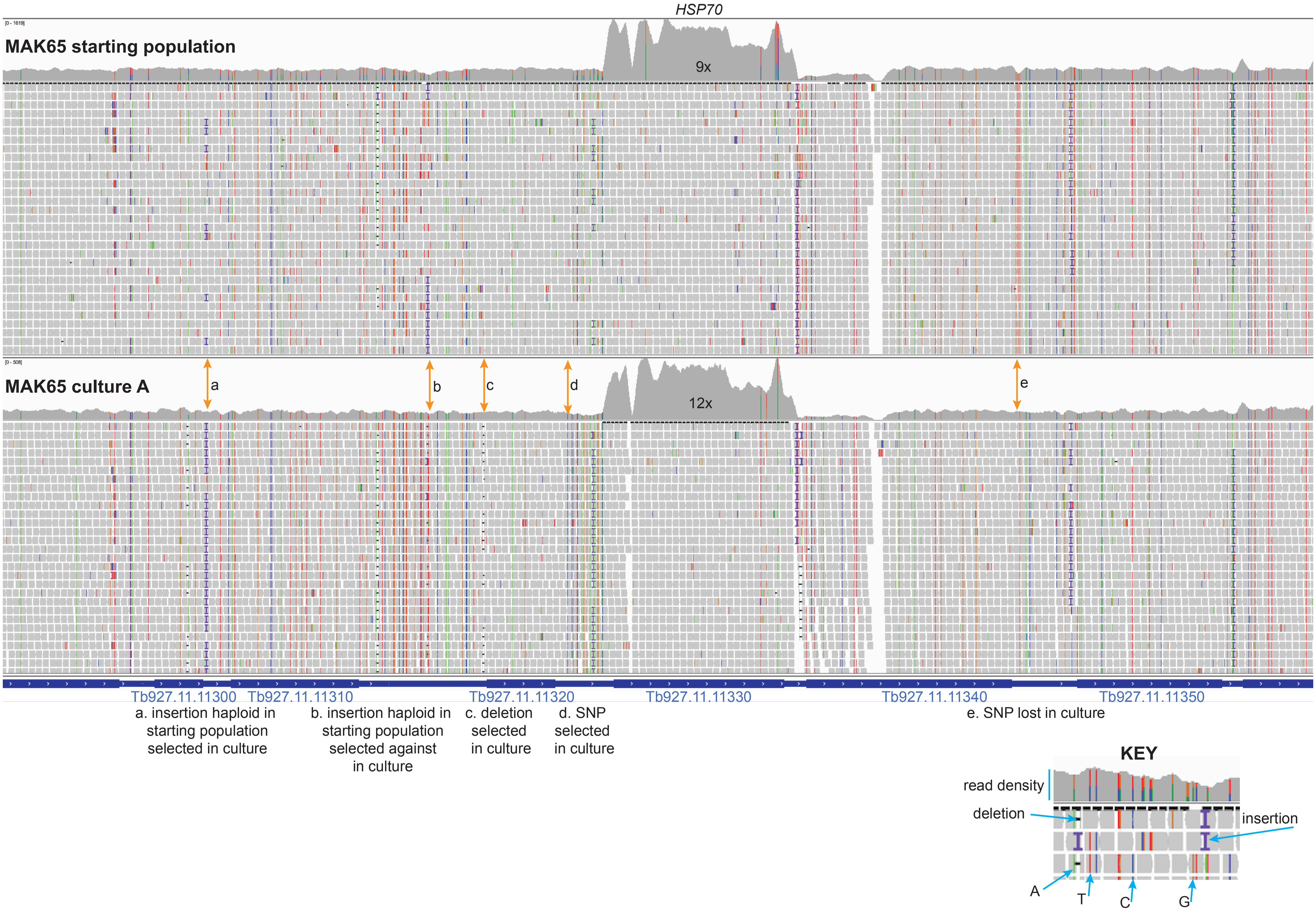
HSP70
[0 - 1619]
MAK65 starting population
9x
[0 - 508]
MAK65 culture A
12x
a
b
c
d
e
Tb927.11.11300
Tb927.11.11310
Tb927.11.11320
Tb927.11.11330
Tb927.11.11340
Tb927.11.11350
a. insertion haploid in starting population selected in culture
b. insertion haploid in starting population selected against in culture
c. deletion selected in culture
d. SNP selected in culture
e. SNP lost in culture
KEY
read density
deletion
insertion
A
T
C
G
